# Marine Bacterial Sialyltransferases

**DOI:** 10.3390/md8112781

**Published:** 2010-11-05

**Authors:** Takeshi Yamamoto

**Affiliations:** Glycotechnology Business Unit, Japan Tobacco Inc., 700 Higashibara, Iwata, Shizuoka 438-0802, Japan; E-Mail: takeshi.yamamoto@jt.com; Tel.: +81-538-32-7389; Fax: +81-538-32-6046

**Keywords:** marine bacteria, *Photobacterium*, sialyltransferase, sialic acid

## Abstract

Sialyltransferases transfer *N*-acetylneuraminic acid (Neu5Ac) from the common donor substrate of these enzymes, cytidine 5′-monophospho-*N*-acetylneuraminic acid (CMP-Neu5Ac), to acceptor substrates. The enzymatic reaction products including sialyl-glycoproteins, sialyl-glycolipids and sialyl-oligosaccharides are important molecules in various biological and physiological processes, such as cell-cell recognition, cancer metastasis, and virus infection. Thus, sialyltransferases are thought to be important enzymes in the field of glycobiology. To date, many sialyltransferases and the genes encoding them have been obtained from various sources including mammalian, bacterial and viral sources. During the course of our research, we have detected over 20 bacteria that produce sialyltransferases. Many of the bacteria we isolated from marine environments are classified in the genus *Photobacterium* or the closely related genus *Vibrio*. The paper reviews the sialyltransferases obtained mainly from marine bacteria.

## 1. Introduction

Sialic acids are important components of carbohydrate chains and are usually found on the terminal position of the carbohydrate moiety of glycoconjugates, including glycoproteins and glycolipids [1,2]. Many studies have shown that the sialyloligosaccharides of glycoconjugates play significant roles in many biological processes, such as inflammatory and immunological responses, cell–cell recognition, cancer metastasis and viral infection [3–7]. The transferring of sialic acids to carbohydrate chains is performed by specific sialyltransferases in the cell [2,8]. Therefore, the sialyltransferases are considered to be key enzymes in the biosynthesis of sialylated glycoconjugates. Detailed investigations of the biological functions of sialylated-glycoconjugates require an abundant supply of the target compounds. Chemical and enzymatic glycosylation have been the two major routes for the preparation of sialylated-glycoconjugates. Although chemical glycosylation has the advantage of high flexibility and wide applicability, the reaction processes are complicated as the chemical reactions often require multiple protection and de-protection steps [9–11]. On the other hand, enzymatic glycosylation using glycosyltransferases is a single-step process with high positional and anomer selectivity and high reaction yield. For example, in sialylation, the transfer of Neu5Ac by sialyltransferases from CMP-Neu5Ac to the appropriate substrate can be readily achieved under mild reaction conditions [12]. Generally, bacterial enzymes are more stable and productive in *Escherichia coli* protein expression systems than the mammalian-derived enzymes, and mammalian enzymes have stricter acceptor specificity. Therefore, we have screened many marine bacteria for novel sialyltransferase activity to enzymatically produce sialylated glycoconjugates and sialyloligosaccharides in large amounts. During the course of our research, we have identified over 20 bacteria that produce sialyltransferases, many of which are classified in the genus *Photobacterium* or the closely related genus *Vibrio* [13]. Furthermore, we have also revealed that the bacteria-derived sialyltransferases show broader acceptor substrate specificity than the mammalian enzymes [13]. These advantages highlight the capacity of bacterial enzymes to serve as efficient tools for the *in vitro* enzymatic modification or/and synthesis of sialylated-glycoconjugates and sialyloligosaccharides [13].

## 2. Sialic Acid

### 2.1. Structure and distribution

Sialic acids (Sias) are a family of monosaccharides comprising over 50 naturally occurring derivatives of neuraminic acid, 5-amino-3,5-dideoxy-d-*glycero*-d-*galacto*-2-nonulosonic acid (Neu) [1,2]. Structurally, the Sia derivatives of Neu carry a variety of substitutions at the amino and/or hydroxyl groups. The amino acid group is often acetylated, glycolylated, or replaced by a hydroxyl group. The hydroxyl groups can be acetylated at O4, O7, O8, or O9, singly or in combination [2], and can also be modified by acetate, lactate, phosphate or sulfate esters.

**Figure 1:**
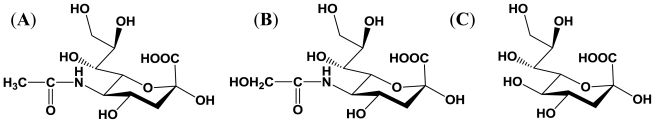
Structures of major three sialic acids. (**A**) *N*-acetylneuraminic acid (Neu5Ac); (**B**) *N*-glycolylneuraminic acid (Neu5Gc); (**C**) 2-keto-3-deoxy-d-*glycero*-d-*galacto*-noninic acid (KDN).

The three major members of the Sia group are *N*-acetylneuraminic acid (Neu5Ac), *N*-glycolylneuraminic acid (Neu5Gc), and 2-keto-3-deoxy-d-*glycero*-d-*galacto*-nonulosonic acid (KDN) [1,2]. Although, Sias are widely distributed in higher animals and some microorganisms, only Neu5Ac is ubiquitous, and Neu5Gc is not found in bacteria [2]. Usually, Sias exist in the carbohydrate moiety of glycoconjugates, and are linked to the terminal positions of the carbohydrate chains of the glycoconjugates [2]. The structures of three major sialic acids are described in Figure 1.

### 2.2. Importance of sialyloligosaccharides

In the sialylated glycoconjugates, mainly four linkage patterns, including Neu5Acα2-6Gal, Neu5Acα2-3Gal, Neu5Acα2-6GalNAc, and Neu5Acα2-8Neu5Ac, are found [1,2]. These structures are formed by specific sialyltransferases in the cell. As described above, sialyloligosaccharides of glycoconjugates play important roles in many biological processes. For example, the relationship between the structure of carbohydrate chains and the recognition of the host cell by influenza virus is one of the best understood biological phenomena. Many studies have shown that influenza A and B viruses bind to cell surface receptors of host cells via Neu5Ac-linked glycoproteins or glycolipids through viral hemagglutinin recognition of host cell Neu5Ac [14–16]. Furthermore, it has been clearly demonstrated that these influenza viruses also recognize the carbohydrate chain structure of the host cell [15]. For example, the avian influenza viruses recognize Neu5Acα2-3Galβ1-3/4GlcNAc structures, and the human influenza viruses recognize Neu5Acα2-6Galβ1-3/4GlcNAc structures [14,15]. The host cell specificity of the influenza viruses is determined mainly by the linkage of Neu5Ac to the penultimate galactose residues and core structures. Thus, the distribution of Neu5Ac and its linkage patterns on the host cell surface are important determinants of host tropism [17,18].

## 3. Sialyltransferase

### 3.1. Classes and sources sialyltransferases

To date, many sialyltransferases have been obtained from mammals, bacteria and viruses [8,19,20]. They are classified into four families according to the carbohydrate linkages they synthesize: the beta-galactoside α2,3-sialyltransferases (ST3Gal I–VI), the beta-galactoside α2,6-sialyltransferases (ST6Gal I, II), the GalNAc α2,6-sialyltransferases (ST6GalNAc I–VI), and the α2,8-sialyltransferases (ST8Sia I–VI) [8]. On the other hand, all the sialyltransferases have been also classified into five families on the basis of sequence similarities in the CAZy (carbohydrate-active enzymes) database [21], *i.e.*, glycosyltransferase family (GT) 29, various sialyltransferases from eukaryotes and viruses; family 38, polysialyltransferase from bacteria such as *Escherichia coli*; family 42, lipooligosaccharide α2,3-sialyltransferase from bacteria such as *Campylobacter jejuni*; family 52, α2,3-sialyltransferase from bacteria such as *Neisseria gonorrhoeae*; and family 80, α2,6-sialyltransferase from marine bacteria such as *Photobacterium damselae*.

It has been revealed that mammalian sialyltransferases contain conserved sequence motifs, including sialyl motifs L, S, VS and motif III [22–24]. Very recently, a crystal structure of the mammalian sialyltransferase was reported [25]. Some functions of these motifs have been clarified. For example, mutagenesis studies have demonstrated that motif L is involved in donor substrate binding [26] and that motif S is involved in donor and acceptor substrate binding [22]. Like the mammalian sialyltransferases, viral α2,3-sialyltransferase, obtained from myxoma-virus-infected RK13 cells, contains the motifs L and S in its catalytic domain and is closely related to mammalian α2,3-sialyltransferase (ST3Gal IV) on the basis of amino acid sequence similarity [20]. The substrate specificity of the viral and mammalian sialyltransferases is very different, despite the common presence of the sialyl motifs.

The known bacterial sialyltransferases do not contain the sialyl motifs found in mammalian sialyltransferases [19,20]. Two short motifs, referred to as the D/E-D/E-G and HP motifs, have been recently identified in the bacterial sialyltransferases and are shown to be functionally important for enzyme catalysis and donor substrate binding [27]. These two motifs are structurally distinct from the motifs found in mammalian sialyltransferases. Furthermore, it has been also demonstrated that the three conserved functional motifs, named YDDGS motif, FKGHP motif and SS motif, exist in the bacterial sialyltransferases that have been classified into GT family 80 [28].

### 3.2. Classification of sialyltransferases produced by bacteria

Genes encoding sialyltransferases have been cloned from various types of bacteria, including *N. gonorrhoeae* [29], *Neisseria meningitidis* [30], *C. jejuni* [31], *E. coli* [32], *P. damselae* [33], *Photobacterium phosphoreum* [34], *Photobacterium leiognathi* [35,36], *Photobacterium* sp. [37], *Vibrio* sp. [38], *Pasteurella multocida* [39], *Haemophilus influenzae* [40], and *Streptococcus agalactiae* [41]. As described above, all bacterial sialyltransferases are classified into four families in the CAZy database: (1) glycosyltransferase (GT) family 38 (polysialyltransferase from *E. coli* and *N. meningitidis*); (2) GT family 42 (lipooligosaccharide α2,3-sialyltransferase and α2,3-/α2,8-sialyltransferase from *C. jejuni* and *H. influenzae*); (3) GT family 52 (α2,3-sialyltransferase from *H. influenzae*, *N. gonorrhoeae*, and *N. meningitidis*); and (4) GT family 80 (α2,6-sialyltransferase and α2,3-/α2,6-sialyltransferase from *P. damselae* and *P. multocida*). Some marine bacterial sialyltransferases also show both sialyltransferase and neuraminidase activities. The relationship between the marine bacterial sialyltransferases and type of enzyme activities is listed in Table 1.

Recently, it has been also reported that marine bacteria producing α2,6-sialyltransferases express Neu5Ac residue on their cell surfaces [42]. Among the bacteria producing α2,6-sialyltransferases, *P. damselae* is known as a causative agent of wound infection, ulceration and pasteurellosis [43–45]. As described in the introduction, sialyloligosaccharides are important molecules for cell–cell interaction and infection. Although the structures of the sialyloligosaccharides produced by the bacteria are still unknown, the molecules containing Neu5Ac on their cell surfaces that might be produced by the enzymes may be involved in virulence and adhesion to host cells.

## 4. Enzymatic Properties of Marine Bacterial Sialyltransferases

### 4.1. An α2,6-sialyltransferase produced by *P. damselae* JT0160

**Figure 2:**
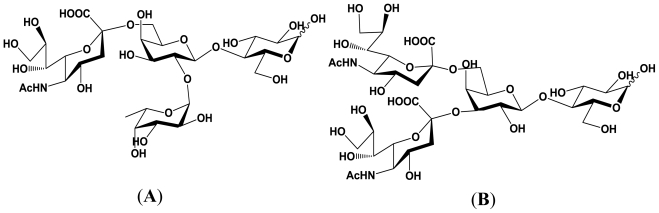
Structures of the enzymatic reaction products derived from 2′-fucosyllactose and 3′-sialyllactose. (**A**) Reaction product derived from 2′-fucosyllactose; (**B**) Reaction product derived from 3′-sialyllactose.

To date, it has been demonstrated that the α2,6-sialyltransferase produced by *P. damselae* JT0160 has unique acceptor substrate specificity compared with the mammalian counterparts. First, it has been revealed that *P. damselae* α2,6-sialyltransferase transfers Neu5Ac to both glycoproteins including asialo-*N*-linked and asialo-*O*-linked glycoproteins and glycolipids [46,47]. However, rat liver α2,6-sialyltransferase has a *K_m_* value approximately 33-times higher for lactose than for *N*-acetyllactosaminide, *P. damselae* α2,6-sialyltransferase recognizes lactose and *N*-acetyllactosaminide as acceptor substrates with almost equal *K_m_* values [48]. These result indicates that *P. damselae* α2,6-sialyltransferase does not recognize the 2-acetamido group in the *N*-acetylglucosaminyl residue, unlike the mammalian enzymes. The acceptor substrate specificity of mammalian sialyltransferases is generally high, and mammalian enzymes are specific for the type of the sugars and linkages. For example, mammalian sialyltransferases do not recognize fucosylated carbohydrate chains as an acceptor substrate. In eukaryotes, the carbohydrate chain moieties of glycoconjugate are synthesized by a series of glycosyltransferases, and the order of glycosylation is determined strictly. Usually, fucosylation is performed by fucosyltransferases after sialylation in mammalian cell. Thus, mammalian sialyltransferases do not recognize fucosylated carbohydrate chain as acceptor substrates and cannot transfer Neu5Ac to them. However, *P. damselae* α2,6-sialyltransferase does recognizes fucosylated carbohydrate chains as an acceptor substrate and transfers Neu5Ac to the galactose residue of carbohydrate chains at position 6 in 2′-fucosyllactose [49]. In addition, the α2,6-sialyltransferase also recognizes 3′-sialyllactose and gives 3′,6′-disialyllactose as the reaction product [49]. The structures of the reaction products derived from 2′-fucosyllactose and 3′-sialyllactose are described in Figure 2.

**Figure 3:**
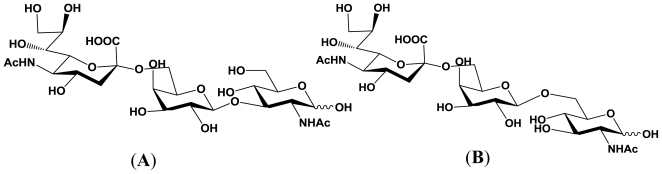
Structures of the enzymatic reaction products derived from Galβ1,3GlcNAc and Galβ1,6GlcNAc. (**A**) Reaction product derived from Galβ1,3GlcNAc; (**B**) Reaction product derived from Galβ1,6GlcNAc.

The *P. damselae* α2,6-sialyltransferase is less specific, which may allow various sialylated glycans to be prepared, as described above. Furthermore, it has been revealed that the α2,6-sialyltransferase uses both Galβ1,3GlcNAc and Galβ1,6GlcNAc as acceptor substrates and gives the corresponding enzymatic reaction products, respectively [50]. These results indicate that this α2,6-sialyltransferase is not sensitive to the nature of the second sugar from the non-reducing terminus and to the linkage between the terminal two sugars. The structures of the reaction products derived from Galβ1,3GlcNAc and Galβ1,6GlcNAc are described in Figure 3.

It has been also revealed that the α2,6-sialyltransferase has unique donor substrate specificity. The sialyltransferase recognizes CMP-Neu5Ac, CMP-KDN as donor substrates and many CMP-sialic acid derivatives with the non-natural modification of an azido or acetylene group at positions C5, C7, C8, and/or C9 [51,52].

### 4.2. An α2,6-sialyltransferase produced by *Photobacterium* sp. JT-ISH-224

**Figure 4:**
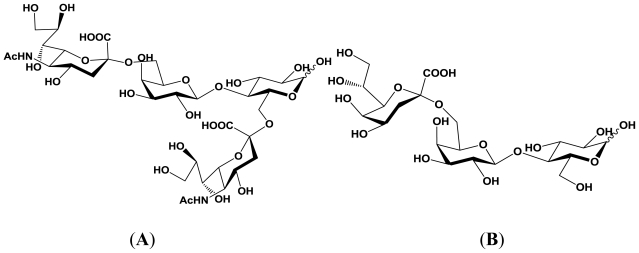
Structures of enzymatic reaction products produced by the α2,6-sialyltransferase cloned from JT-ISH-224. (**A**) 6,6′-sisialyllactose; (**B**) KDO-lactose.

Except α2,6-sialyltransferase produced by *P. damselae* JT0160, three additional genes encoding the marine bacterial α2,6-sialyltransferase have been cloned from *Photobacterium* sp. JT-ISH-224 [37], *P. leiognathi* JT-SHIZ-145 [35] and *P. leiognathi* JT-SHIZ-119 [36]. These genes have all been expressed as recombinant active form enzymes in an *E. coli* protein expression system. Among them, α2,6-sialyltransferase cloned from *Photobacterium* sp. JT-ISH-224 has been shown to display the highest specific activity. A homology search shows that the amino acid sequence of the α2,6-sialyltransferase from JT-ISH-224 has 54% identity to α2,6-sialyltransferase cloned from *P. damselae* JT0160. The enzymatic property of the α2,6-sialyltransferase cloned from *Photobacterium* sp. JT-ISH-224 is similar to that of the α2,6-sialyltransferase cloned from *P. damselae* JT0160. To date, it has been revealed that the α2,6-sialyltransferase cloned from JT-ISH-224 can transfer Neu5Ac to both O-6 and O-6′ hydroxyl groups of lactose simultaneously and gives 6,6′-disialyllactose, and transfers KDO (2-keto-3-deoxyoctonate) to the O-6′ hydroxyl group of lactose and gives KDO-lactose [53]. The structures of the reaction products described here are shown in Figure 4.

### 4.3. An α2,3-sialyltransferase produced by *P. phosphoreum* JT-ISH-467

**Figure 5:**
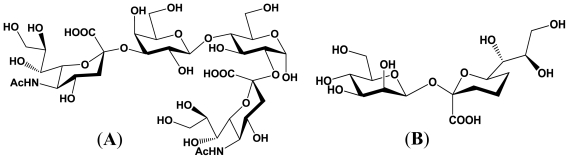
Structures of enzymatic reaction products produced by the α2,3-sialyltransferase cloned from JT-ISH-224. (**A**) Enzymatic reaction product derived from lactose; (**B**) Enzymatic reaction product derived from mannose.

To date, three genes encoding the marine bacterial α2,3-sialyltransferase have been cloned from *Photobacterium* sp. JT-ISH-224 [37], *P. phosphoreum* JT-ISH-467 [34] and *Vibrio* sp. JT-FAJ-16 [38] and all these genes have been expressed as recombinant active form enzymes in an *E. coli* protein expression system. During the course of 3′-sialyllactose production using α2,3-sialyltransferase cloned from *Photobacterium* sp. JT-ISH-224, it has been revealed that the α2,3-sialyltransferase can transfer Neu5Ac to both O-2 and O-3′ hydroxyl groups of lactose simultaneously, giving 2,3′-disialyllactose as an enzymatic reaction product [54]. By NMR spectroscopy analysis, it has been confirmed that the reaction product contain only α-form. The relative configuration between C-1 to C-3 of the α-glucopyranose residue is superimposable with that between C-4 to C-2 of galactopyranose. Therefore, it is expected that the enzyme recognizes the α-glucopyranose residue as acceptor substrate and transfers Neu5Ac to O-2 hydroxyl group of the α-glucopyranose. In addition, it has been also demonstrated that the α2,3-sialyltransferase can transfer Neu5Ac to the β-anomeric hydroxyl groups of mannose and gives the corresponding reaction product [50]. In this case, the stereochemistry of β-mannose residue from C-2 to O-5 in the pyranose ring is superimposable on that from C-4 to C-2 of galactopyranose, except for the difference between the C-2 carbon atom in the Gal and O-5 oxygen atom in the Man [50]. Thus, it is strongly expected that the α2,3-sialyltransferase recognizes the acceptor substrate mainly through the stereochemical structure of the C-4 to C-3 of Gal [50]. The structures of the reaction products produced by the enzyme are shown in Figure 5.

**Figure 6:**
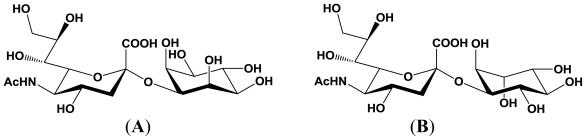
Structures of the enzymatic reaction products derived from *epi*-inositol and 1_D_-*chiro*-inositol. (**A**) Reaction product derived from *epi*-inositol; (**B**) Reaction product derived from 1_D_-*chiro*-inositol.

Very recently, it has been also reported that this α2,3-sialyltransferase can catalyze the transfer of Neu5Ac residue to inositols, non-carbohydrates, as well as to carbohydrates with a diol structure corresponding to the C-3 and C-4 of the galactopyranose moiety. As described, the α2,3-sialyltransferase does recognizes both *epi*-inositol and 1_D_-*chiro*-inositol as acceptor substrates, and gives the corresponding reaction products [55]. The structures of the reaction products derived from *epi*-inositol and 1_D_-*chiro*-inositol are described in Figure 6.

## 5. Application of Sialyltransferases

### 5.1. Production of sialyloligosaccharides

A remarkable oligosaccharide production method has been reported using a single growing metabolically engineered bacterium [56]. In this method, oligosaccharides are produced in a bacterium that overexpresses the recombinant glycosyltransferase genes and that maintains the pool level of the sugar nucleotides by its enzymatic cellular machinery [57,58]. To date, it has been reported that several oligosaccharides, such as lacto-*N*-neotetraose, lacto-*N*-neoheaxose, and sialyllactose, are efficiently produced by this method using bacterial glycosyltransferases, such as β1,3-*N*-acetylglucosaminyltransferase, β1,4-galactosyltransferase and α2,3-sialyltransferase from *N. meningitidis*, α1,2-fucosyltransferase from *Helicobacter pylori*, *etc.*[56–58]. Very recently, it has been also reported that more than 25 g/L of both 6′-sialyllactose and 3′-sialyllactose have been produced by a high-density cell culture of *E. coli* strains overexpressing bacterial sialyltransferases of α2,6-sialyltransferase from *Photobacterium* sp. and α2,3-sialyltransferase from *N. meningitidis*, respectively [53,59]. In the case of the sialyllactose production system using the transformed bacterium by *Photobacterium* sp. JT-ISH-224 α2,6-sialyltransferase gene, formation of KDO-lactose and 6,6′-disialyllactose has been observed as well as 6′-sialyllactose production [53]. On the other hand, no formation of by-product has been observed in case of sialyllactose production system using the transformed bacterium by the *N. meningitidis* α2,3-sialyltransferase gene. These results indicate that the 6′-sialyllactose production system with marine bacterial sialyltransferase has the possibility to produce various kinds of sialylated carbohydrate chains, but examination of the culture conditions are necessary to efficiently produce the compounds aimed for [53].

### 5.2. Application of sialyloligosaccharides

As described above, influenza A and B viruses infect host cells through the binding of viral hemagglutinins to sialylglycoproteins or sialylglycolipids of the receptors on the host cell surface [7,14–18]. These influenza viruses recognize both sialic acid in the receptor on the cell surface and carbohydrate chain structures on the cell surface including Neu5Acα2-3(6)Galβ1-3GlcNAc (sialyl-lacto series Type I) or Neu5Acα2-3(6)Galβ1-4GlcNAc (sialyl-lacto series Type II). Therefore, molecules with the carbohydrate chain structures described above are thought to be potential inhibitors of infection by influenza virus or to be materials for influenza virus adsorption [60]. In addition, the ability of influenza viruses to recognize sialyloligosaccharides is known to be enhanced by the clustering of sialyloligosaccharides on macromolecules [61]. Thus, a variety of glycopolymers with multivalent sialyloligosaccharides that target influenza viral hemagglutinines have been synthesized for developing influenza virus inhibitors or adsorption materials [60]. Up to present, various glycopolymers carrying sialyloligosaccharides have been produced using polyacrylamide, γ-polyglutamic acid, polystyrene, chitosan and dendrimers as the polymer backbone, and their inhibition of influenza virus infection has also been demonstrated [60–68].

Recently, it has been clearly demonstrated that sialyllactose (Neu5Acα2-3(6)Galβ1-4Glc), Neu5Ac and *N*-acetylmannosamine could be candidates for prophylactic drugs to distal myopathy with rimmed vacuoles (DMRV)-hereditary inclusion body myopathy (hIBM), which is a moderately progressive autosomal recessive myopathy [69]. The disease gene underlying DMRV-hIBM has been predicted to be GNE, encoding UDP-*N*-acetylglucosamine-2-epimerase and *N*-acetylmannosamine kinase [70,71]. These enzymes are essential for sialic acid biosynthesis. Thus, the patients who have this disease show hyposialylation in various organs [72]. A prophylactic treatment with sialyllactose, Neu5Ac and *N*-acetylmannosamine was tested to confirm effectiveness in a mouse model of DMRV-hIBM. Oral treatment with the sialic acid metabolites described above completely precluded the development of the myopathic phenotype in the model mice [72]. Therefore, it is strongly expected that DMRV-hIBM in humans can potentially be treated by oral addition of sialic acid metabolites, such as sialyllactose, Neu5Ac and *N*-acetylmannosamine, and their derivatives.

## Figures and Tables

**Table 1:** The relationship between marine bacteria and enzyme activities.

Enzyme origin	Enzyme activities	
	Sialyltransferase activity	Neuraminidase activity
*Photobacterium phosphoreum* JT-ISH-467	α2,3-sialyltransferase	+
*Photobacterium* sp. JT-ISH-224		+
*Vibrio* sp. JT-FAJ-16		−
*Photobacterium damselae* JT0160	α2,6-sialyltransferase	−
*Photobacterium leiognathi* JT-SHIZ-119		+
*Photobacterium leiognathi* JT-SHIZ-145		−
*Photobacterium* sp. JT-ISH-224		−
